# TyG-BMI and risk of CKD progression: Mediation by oxidative stress, fibrosis, and metabolic dysfunction

**DOI:** 10.1097/MD.0000000000047465

**Published:** 2026-02-28

**Authors:** Bin Zhao, YunFei Guo, Yuyang Liu, Xue Zhou, Ping Han, Gang Fu, Shanshan Guo, FuChun Zhang, Yuzhu Wang

**Affiliations:** a Department of Nephrology, Beijing Haidian Hospital (Haidian Section of Pecking University Third Hospital), Beijing, China; b Center for Epidemiology, Institute of Medical Information, Chinese Academy of Medical Sciences and Peking Union Medical College, Beijing, China; c Department of Nephrology, Haihe Hospital Tianjin, China.

**Keywords:** cardiovascular-kidney-metabolic, fibrosis-4 index (FIB-4), mediation analysis, metabolic dysfunction, triglyceride-glucose body mass index (TYG-BMI), uric acid-to-HDL ratio (UHR)

## Abstract

To investigate the association between the triglyceride-glucose-body mass index (TyG-BMI) and chronic kidney disease (CKD) progression in individuals across the cardiovascular-kidney-metabolic spectrum, and to evaluate the potential mediation by biomarkers of oxidative stress, inflammation, and fibrosis. This study included 9265 adults stratified into cardiovascular-kidney-metabolic stages 0 to 4. Logistic regression and restricted cubic splines assessed the relationship between TyG-BMI and CKD progression. Mediation analysis quantified the indirect effects of the uric acid-to-high-density lipoprotein ratio, fibrosis-4 index, and red cell distribution width-to-albumin ratio. The primary finding of this study is that elevated TyG-BMI significantly increases the risk of chronic kidney disease (CKD) progression. Specifically, for each unit increase in TyG-BMI, the risk of CKD progression rose by 16% (OR = 1.16; 95% CI:1.01–1.30). This association was quantitatively and significantly mediated by the uric acid-to-high-density lipoprotein ratio, the fibrosis-4 index, and the red cell distribution width-to-albumin ratio, indicating the involvement of oxidative, fibrotic, and inflammatory pathways. Our analysis suggests that elevated TyG-BMI may be associated with an increased risk of CKD progression, potentially mediated through oxidative, inflammatory, and fibrotic pathways. These findings warrant further investigation to confirm the potential clinical relevance of TyG-BMI and the implicated pathways.

## 1. Introduction

Chronic kidney disease (CKD) represents a major global health challenge, affecting 843 million people worldwide. Within the cardiovascular-kidney-metabolic (CKM) syndrome spectrum, nearly 30% of adults over 40 are in a pre-CKD state, facing significantly elevated risks of cardiovascular mortality and dialysis initiation.^[1-5]^

The triglyceride-glucose-body mass index (TyG-BMI), a composite indicator of insulin resistance, has been closely associated with CKD progression.^[6-9]^ However, the specific mechanisms through which it contributes to renal injury remain unclear. We hypothesize that TyG-BMI may impair renal function through multiple pathways, including uric acid metabolism dysregulation (uric acid-to-high-density lipoprotein ratio [UHR]), liver fibrosis (FIB-4), and systemic inflammation (RAR).^[6,10-14]^

To test this hypothesis, we utilized data from the National Health and Nutrition Examination Survey (NHANES) and applied structural equation modeling to analyze the association between TyG-BMI and incident CKD, while evaluating the mediating effects of these biomarkers. This study aims to clarify the pathogenesis of metabolic nephropathy and provide new targets for early intervention.

## 2. Materials and methods

### 2.1. Study population and data source

Data for this analysis were derived from the NHANES, a nationally representative, cross-sectional surveillance program conducted by the National Center for Health Statistics. NHANES employs a complex, multistage, stratified probability sampling design to select participants representative of the noninstitutionalized civilian US population. The survey methodology includes structured household interviews, standardized physical examinations, and laboratory testing at mobile examination centers. The study protocol was approved by the National Center for Health Statistics Institutional Review Board, and written informed consent was obtained from all participants. Publicly available data from 6 consecutive survey cycles (2007–2018) were accessed through via the NHANES online repository (https://wwwn.cdc.gov/nchs/nhanes/search/; Table S1, Supplemental Digital Content, https://links.lww.com/MD/R413).

### 2.2. Participant selection and CKM staging

**Figure 1.**
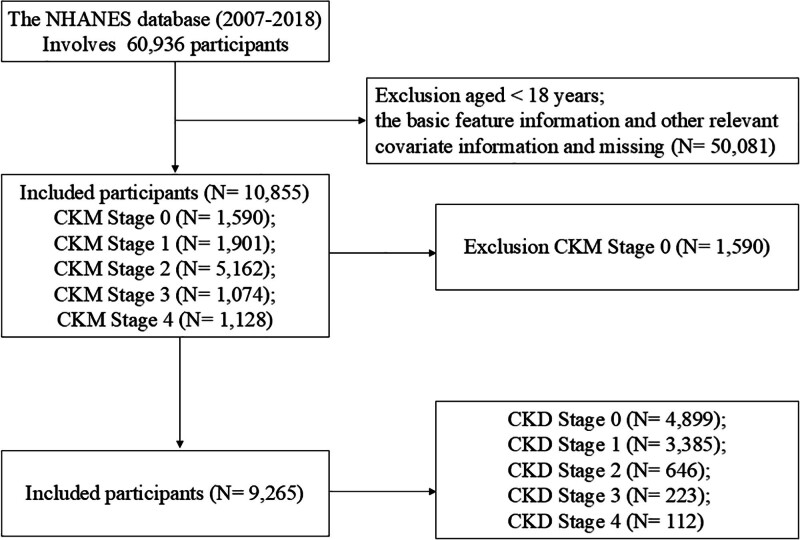
Flow chart of the participants’ selection. CKD = chronic kidney disease, CKM = cardiovascular-kidney-metabolic, GED = general educational development, NHANES = National Health and Nutrition Examination Survey

From an initial cohort of 60,936 participants, we applied the following exclusion criteria: age < 18 years, pregnancy, and missing data for critical variables including TyG-BMI, biomarkers (UHR, FIB-4, RAR), kidney function parameters, or essential covariates. The final analytical sample comprised 10,855 adults, who were classified into 5 CKM stages according to established criteria: stage 0 (no risk factors, n = 1590), stage 1 (adipose tissue dysfunction/prediabetes, n = 1901), stage 2 (metabolic dysregulation/chronic kidney disease, n = 5162), stage 3 (subclinical cardiovascular disease, n = 1074), and stage 4 (clinical cardiovascular disease, n = 1128). To account for the complex survey design and produce nationally representative estimates, we incorporated appropriate survey weights, stratification variables, and clustering terms in all analyses. The dataset was randomly partitioned into training and validation sets at a 7:3 ratio for internal validation of findings. CKD status was defined according to the 2012 Kidney Disease Improving Global Outcomes Clinical Practice Guidelines, stratifying participants into non-CKD and CKD groups (Fig. 1 and Table 1).

**Table 1:** Characteristics of study participants.

Characteristics	All (n = 9265)	Normal (n = 4899)	CKD (n = 4366)	*P*-value
Demographic				
Gender, n (%)				**.003**
Man	4693 (50.7)	2414 (49.3)	2279 (52.2)	
Female	4572 (49.3)	2485 (50.7)	2087 (47.8)	
Age (yr)	51.4 ± 17.4	41.7 ± 13.7	62.3 ± 14.3	**<.001**
Education level, n (%)				**<.001**
<9th grade	964 (10.4)	530 (10.8)	434 (9.9)	
9-11th grade	1383 (14.9)	799 (16.3)	584 (13.4)	
High school graduate/GED or equivalent	4336 (46.8)	1939 (39.6)	2397 (54.9)	
Some college or AA degree	1839 (19.8)	771 (15.7)	1068 (24.5)	
College graduate or above	687 (7.4)	447 (9.1)	240 (5.5)	
PIR, n (%)				**<.001**
<1.3	2866 (30.9)	1727 (35.3)	1139 (26.1)	
≥1.3&<3.5	3545 (38.3)	1804 (36.8)	1741 (39.9)	
≥3.5	2854 (30.8)	1368 (27.9)	1486 (34.0)	
Alcohol, n (%)	2579 (27.8)	1235 (25.2)	1344 (30.8)	**<.001**
Smoking, n (%)	4871 (52.6)	2700 (55.1)	2171 (49.7)	**<.001**
Hypertension, n (%)	5456 (58.9)	2266 (46.3)	3190 (73.1)	**<.001**
Diabetes, n (%)				**<.001**
1	1266 (13.7)	499 (10.2)	767 (17.6)	
2	7761 (83.8)	4301 (87.8)	3460 (79.2)	
3	238 (2.6)	99 (2.0)	139 (3.2)	
**Depression, n (%**)	**794 (8.6**)	**444 (9.1**)	**350 (8.0**)	**.039**
**TyG-BMI, median (IQR**)	**121 (103, 146**)	**122 (103, 146**)	**121 (103, 145**)	**.039**
**UHR, median (IQR**)	**10.85 (8.00, 14.40**)	**10.6 (7.73, 14.00**)	**11.12 (8.37, 14.87**)	**.016**
**FIB-4, median (IQR**)	**1.03 (0.68, 1.53**)	**0.78 (0.54, 1.10**)	**1.39 (1.01, 1.91**)	**<.001**
**RAR, median (IQR**)	**3.09 (2.88, 3.37**)	**3.05 (2.84, 3.33**)	**3.15 (2.91, 3.40**)	**<.001**

Bold values indicate statistical significance.

BMI = body mass index, CKD = chronic kidney disease, FIB-4 = fibrosis-4 index, PIR = family poverty-income ratio, RAR = red cell distribution width-to-albumin ratio, UHR = uric acid-to-high-density lipoprotein ratio.

### 2.3. Exposure variables

The TyG-BMI is a validated surrogate marker of insulin resistance that integrates metabolic and obesity-related parameters. It is calculated as the product of TyG and BMI, where TyG is defined as the (natural logarithm [ln] of the product of fasting triglyceride level [triglycerides, mmol/L] and fasting plasma glucose [mg/dL], divided by 2). Higher TyG-BMI values are positively associated with increased cardiometabolic risk and the severity of insulin resistance.^[10]^


$$\begin{array}{l} TyG=\ln\left(\frac{TG\times FPG}{2}\right) \end{array}$$

$$\begin{array}{l} BMI=\frac{Weight\left(kg\right)}{Height{\left(m\right)}^{2}} \end{array}$$

$$\begin{array}{l} TyG-BMI=TyG\times BMI \end{array}$$

The fibrosis-4 index (FIB-4) is a noninvasive biomarker for liver fibrosis, incorporating age, platelet count, alanine aminotransferase (ALT), and aspartate aminotransferase (AST). It is calculated by multiplying platelet count by ALT and dividing by the product of age and AST, with ALT and AST in U/L and platelet count in 10^9^/L. A FIB-4 score < 1.45 suggests minimal fibrosis, scores between 1.45 and 3.25 indicate intermediate probability, and values > 3.25 are highly suggestive of advanced fibrosis or cirrhosis.^[12]^


$$\begin{array}{l} FIB-4=\frac{Age\left(years\right)\times AST\left(U/L\;\right)}{platelet\times \sqrt{ALT\left(U/L\;\right)}} \end{array}$$

The UHR is an emerging marker of metabolic dysfunction and cardiovascular risk. It is computed by dividing serum uric acid (mg/dL) by high-density lipoprotein cholesterol (mg/dL) and multiplying by 100. UHR reflects impaired purine metabolism and disturbed lipid homeostasis.^[15]^

The RAR represents systemic inflammation and hematologic changes. It is calculated as red cell distribution width (%) divided by serum albumin (g/L), serving as an indicator of inflammatory burden and nutritional deficiencies.^[11]^

### 2.4. Outcome and covariates

The outcome variable was the staging of CKM syndrome, defined in accordance with the American Heart Association classification, which includes 5 stages – Stage 0: absence of CKM risk factors; Stage 1: excess or dysfunctional adipose tissue; Stage 2: metabolic risk factors or chronic kidney disease; stage 3: subclinical cardiovascular disease; and Stage 4: clinical cardiovascular disease (Table S2, Supplemental Digital Content, https://links.lww.com/MD/R413).

For analytical purposes, CKM syndrome was dichotomized into a binary variable: stage 0 denoting the absence of CKM syndrome and stages 1 to 4 indicating the presence of CKM syndrome (Tables S4 and S5, Supplemental Digital Content, https://links.lww.com/MD/R413).

A comprehensive set of covariates was included in the analysis, encompassing basic personal information, family background, health behaviors, and social characteristics (Table S3, Supplemental Digital Content, https://links.lww.com/MD/R413). Personal information included sex, age, poverty-income ratio, and education level. Health-related behaviors and clinical markers comprised smoking status, alcohol consumption, triglyceride levels, high-density lipoprotein, low-density lipoprotein, and diabetes status.^[13]^

### 2.5. Statistical analysis

Descriptive statistics were presented as mean ± standard deviation for normally distributed continuous variables, and as median with interquartile range (IQR) for non-normally distributed variables, with the Kruskal–Wallis test applied for group comparisons. Categorical variables were reported as frequencies and percentages. Logistic regression models were employed to estimate odds ratios (ORs) and 95% confidence intervals (CIs) for the association between systemic immune-inflammatory indices and CKD risk, treating the immune-inflammatory indices as both continuous and categorical variables (quartiles based on control group distribution; Q1 used as reference). Exposure-response relationships between systemic immune-inflammatory indices and CKD were assessed using restricted cubic spline functions. Causal mediation analysis was performed under the assumptions of: causal pathways between exposure (TyG-BMI), mediators (UHR, FIB-4, RAR), and outcomes (CKD risks); linearity among variables; and absence of unmeasured confounding factors affecting mediators and outcomes. Indirect effects and their 95% CIs were estimated via 1000 bootstrap resamples. Interaction terms were tested to evaluate effect modification, including the interaction term CKM stage × systemic immune-inflammatory indices. All statistical analyses were conducted using R software, version 4.4.2 (R Foundation for Statistical Computing, Vienna, Austria; https://cran.r-project.org). A 2-sided *P* value < .05 was considered statistically significant.

## 3. Results

### 3.1. Baseline characteristics of study participants

The final analytical cohort included 9265 participants, comprising 4899 without CKD and 4366 with CKD. Participants with CKD were significantly older (62.3 ± 14.3 vs 41.7 ± 13.7 years, *P* < .001) and had a higher proportion of males (52.2% vs 49.3%, *P* = .003). Socioeconomically, educational attainment differed, with fewer CKD participants having completed college (5.5% vs 9.1%), while a lower proportion of CKD individuals lived in extreme poverty (26.1% vs 35.3% with poverty-income ratio < 1.3, *P* < .001).

The CKD group exhibited significantly poorer cardiometabolic profiles, including a higher prevalence of hypertension (73.1% vs 46.3%) and diabetes (17.6% vs 10.2%). Behavioral differences included lower rates of current smoking (49.7% vs 55.1%) and higher prevalence of alcohol consumption (30.8% vs 25.2%) among CKD individuals (both *P* < .001).

Metabolic evaluations revealed a significantly different distribution of TyG-BMI scores in the CKD group (122.4 ± 15.1 vs 119.8 ± 14.3, *P* < .001), alongside evidence of increased liver fibrosis as indicated by FIB-4 (1.39 vs 0.78, *P* < .001). CKD subjects also demonstrated higher UHR (11.12 vs 10.60, *P* = .016) and RAR (3.15 vs 3.05, *P* < .001), despite lower low-density lipoprotein-C levels (112 vs 114 mg/dL, *P* < .001; Table 1).

Multivariable logistic regression identified UHR, FIB-4, and RAR as strong predictors of CKD across progressive adjustment models. UHR showed a strong dose-response, with each unit increase associated with 5% higher CKD risk in the fully adjusted model (OR = 1.05, 95% CI: 1.04–1.06, *P* < .001). Risk increased across quartiles, with Q4 showing the highest risk (OR = 2.06, 95% CI: 1.76–2.42, *P* trend < .001).

FIB-4 showed the strongest association, with each unit increase corresponding to a 52% higher risk of CKD (OR = 1.52, 95% CI: 1.40–1.66, *P* < .001). Participants in Q4 of FIB-4 exhibited an OR of 5.47 (95% CI: 4.47–6.72). RAR also maintained significant associations in adjusted models (per unit OR = 1.39, 95% CI: 1.02–1.27; Q4 OR = 1.37, 95% CI: 1.17–1.58; both *P* < .001). TyG-BMI demonstrated a modest association (Q2 OR = 1.16, 95% CI: 1.01–1.34, *P* = .033; Table 2).

**Table 2:** Association of metabolic indices with CKD.

Indices	Model 1		Model 2		Model 3	
	OR (95%CI)	*P*-Value	OR (95%CI)	*P*-Value	OR (95%CI)	*P*-Value
TyG-BMI						
Per unit	1.00 (0.99, 1.00)	.258	1.00 (0.99, 1.00)	.193	1.00 (0.99, 1.01)	.973
Q1	Reference		Reference		Reference	
Q2	1.16 (1.01, 1.32)	.037	1.13 (0.98, 1.30)	.074	1.16 (1.01, 1.34)	.033
Q3	1.02 (0.89, 1.17)	.761	1.01 (0.88, 1.16)	.890	1.00 (0.87, 1.15)	.962
Q4	1.17 (1.02, 1.35)	.022	1.18 (1.03, 1.36)	.019	1.09 (0.95, 1.26)	.214
*P* trend	1.00 (0.99, 1.00)	.085	1.00 (0.99, 1.00)	.065	1.01 (0.99, 1.01)	.563
UHR						
Per unit	1.05 (1.04. 1.06)	<.001	1.05 (1.04, 1.06)	<.001	1.05 (1.04, 1.06)	<.001
Q1	Reference		Reference		Reference	
Q2	1.37 (1.18, 1.58)	<.001	1.41 (1.22, 1.63)	<.001	1.41 (1.22, 1.63)	<.001
Q3	1.52 (1.31, 1.77)	<.001	1.55 (1.34, 1.81)	<.001	1.52 (1.31, 1.77)	<.001
Q4	2.04 (1.75, 2.39)	<.001	2.12 (1.81, 2.48)	<.001	2.06 (1.76, 2.42)	<.001
*P* trend	1.06 (1.05, 1.08)	<.001	1.07 (1.05, 1.08)	<.001	1.06 (1.05, 1.08)	<.001
FIB-4						
Per unit	1.65 (1.51, 1.80)	<.001	1.59 (1.46, 1.74)	<.001	1.52 (1.40, 1.66)	<.001
Q1	Reference		Reference		Reference	
Q2	2.33 (1.90, 2.86)	<.001	2.19 (1.78, 2.70)	<.001	2.11 (1.72, 2.60)	<.001
Q3	3.44 (2.84, 4.20)	<.001	3.14 (2.58, 3.84)	<.001	2.96 (2.43, 3.63)	<.001
Q4	6.58 (5.41, 8.04)	<.001	5.97 (4.89, 7.32)	<.001	5.47 (4.47, 6.72)	<.001
*P* trend	3.73 (3.28, 4.24)	<.001	3.51 (3.08, 4.00)	<.001	3.29 (2.88, 3.76)	<.001
RAR						
Per unit	1.10 (0.99, 1.22)	.064	1.16 (1.04, 1.29)	<.001	1.39 (1.02, 1.27)	<.001
Q1	Reference		Reference		Reference	
Q2	1.07 (0.92, 1.23)	.373	1.07 (0.93, 1.24)	.360	1.08 (0.93, 1.25)	.302
Q3	1.35 (1.17, 1.56)	<.001	1.38 (1.20, 1.60)	<.001	1.39 (1.20, 1.61)	<.001
Q4	1.30 (1.12, 1.50)	<.001	1.39 (1.20, 1.61)	<.001	1.37 (1.17, 1.58)	<.001
*P* trend	1.37 (1.17, 1.59)	<.001	1.48 (1.27, 1.74)	<.001	1.44 (1.23, 1.69)	<.001

Model 1: adjusted for age, sex, and race.

Model 2: further adjusted for marital status, education level, family poverty-income ratio (PIR), smoking status, and alcohol use.

Model 3: further adjusted drugs for hypertension, diabetes, and depression status.

BMI = body mass index, CI = confidence interval, CKD = chronic kidney disease, FIB-4 = fibrosis-4 index, OR = odds ratio, PIR = family poverty-income ratio, RAR = red cell distribution width-to-albumin ratio, UHR = uric acid-to-high-density lipoprotein ratio.

**Figure 2.**
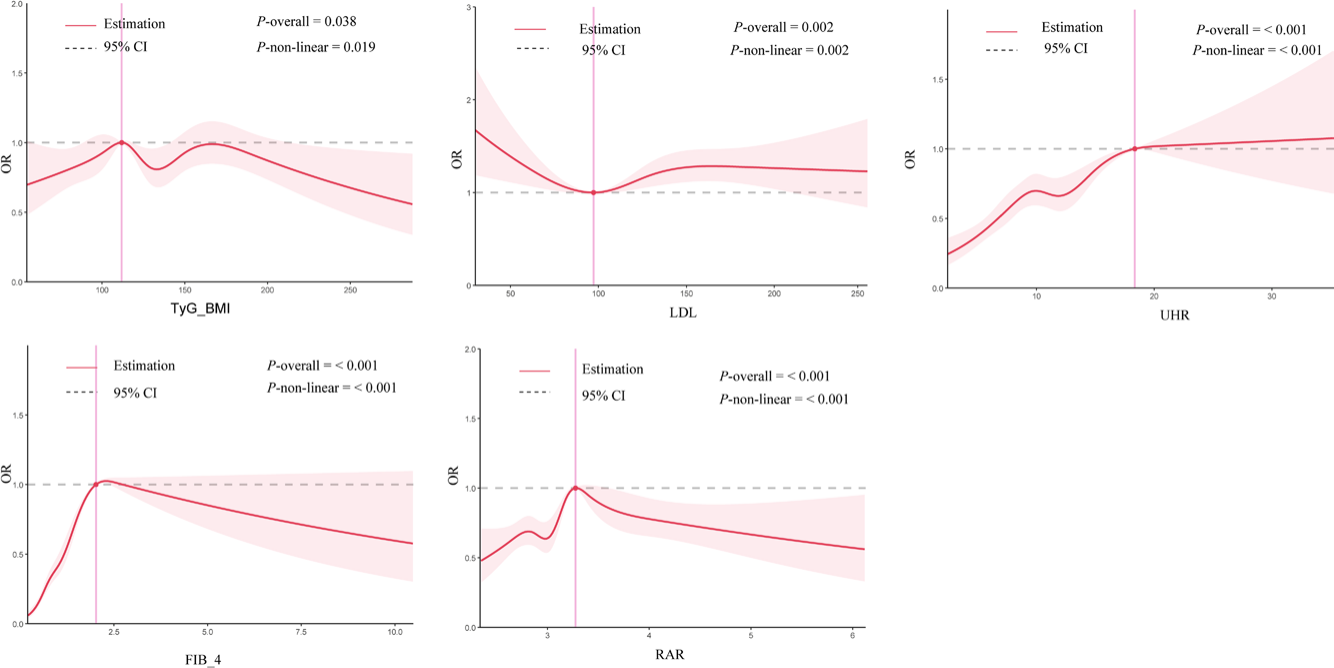
Dose-response relationships between cardiometabolic indices and chronic kidney. The solid line represents the estimated utility values, and the 2 dashed lines represent the 95% confidence interval of the estimated utility values. The nodes are set to 4, corresponding to the 5th, 35th, 65th, and 95th percentiles. The model was adjusted for age, sex, race/ethnicity, systolic blood pressure, smoking status, and history of cardiovascular disease, marital status, education level, family poverty-income ratio (PIR), alcohol use, drugs for hypertension, diabetes, and depression status. BMI = body mass index, CI = confidence interval, FIB-4 = fibrosis-4 index, LDL = low-density lipoprotein, OR = odds ratio, PIR = family poverty-income ratio, RAR = red cell distribution width-to-albumin ratio, UHR = uric acid-to-high-density lipoprotein ratio.

Restricted cubic spline analysis revealed nonlinear associations between CKD and FIB-4 (*P* overall = .038, *P* non-linear = .019), TyG-BMI (*P* overall < .001, *P* non-linear < .001), RAR (*P* overall < .001, *P* non-linear < .001), and UHR (*P* overall < .001, *P* non-linear < .001; Fig. 2).

### 3.2. Associations of TyG-BMI with UHR, RAR, and FIB-4

Multivariable linear regression analyses demonstrated significant associations between TyG-BMI and all 3 metabolic markers (Table S6, Supplemental Digital Content, https://links.lww.com/MD/R413). For UHR, each unit increase in TyG-BMI was associated with higher UHR across models (Model 1: β = 3.58; Model 3: β = 3.41, 95% CI: 3.27–3.55, *P* < .001), demonstrating a stable association throughout the adjustment process. FIB-4 showed a strong negative association (Model 1: β = –2.95 to Model 3: β = –3.75, 95% CI:–4.58 to–2.92, *P* < .001), indicating a higher relationship strength with covariate adjustment. RAR displayed strong positive associations (Model 1: β = 14.4 to Model 3: β = 12.2, 95% CI: 10.7–13.7, *P* < .001), confirming that TyG-BMI has temporal relationships with UHR, FIB-4, and RAR biomarkers throughout the adjustment continuum.

### 3.3. Association of metabolic indices with CKM

Stratified analyses revealed stage-specific effects of metabolic indices across the CKM spectrum. UHR showed a progressive increase in CKD risk with CKM severity with no significant association in stage 1 (OR = 1.00) but progressively elevated CKD risk in stage 2 (OR = 1.04, 95% CI: 1.02–1.05) and 4 (OR = 1.09, 95% CI: 1.05–1.13; *P* = .003). FIB-4 presented a bimodal risk pattern, with peak effects observed at stage 2 (OR = 1.55, 95% CI: 1.39–1.74) and again at stage 4 (OR = 1.65, 95% CI: 1.29–2.17). RAR displayed a transition from neutral (stage 1 OR = 0.83) to elevated risk in advanced disease (stage 4 OR = 1.32, *P* = .091; *P*-interaction = .002), suggesting a dynamic role of inflammation across disease progression. These findings highlight the different mechanistic contributions of metabolic dysregulation, hepatic fibrosis, and renal vascular dysfunction at various stages of CKM, supporting their use in risk stratification and disease monitoring.

### 3.4. Mediating roles of UHR, FIB-4, and RAR in TyG-BMI-associated progression from CKM disorders to CKD

**Figure 3.**
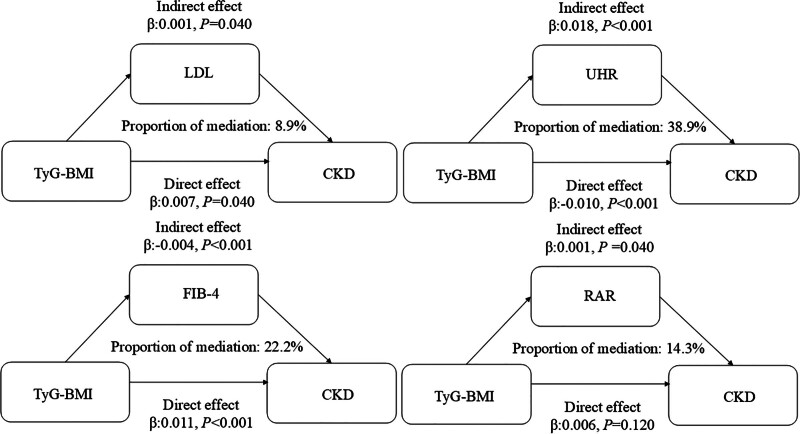
Analysis of the mediating effect. The model was adjusted for age, sex, race/ethnicity, systolic blood pressure, smoking status, and history of cardiovascular disease, marital status, education level, family poverty-income ratio (PIR), alcohol use, drugs for hypertension, diabetes, and depression status. BMI = body mass index, FIB-4 = fibrosis-4 index, LDL = low-density lipoprotein, PIR = family poverty-income ratio, RAR = red cell distribution width-to-albumin ratio, UHR = uric acid-to-high-density lipoprotein ratio.

Causal mediation analysis indicated that TyG-BMI contributes to CKD progression in the CKM spectrum both directly and indirectly via systemic metabolic disturbances. Significant indirect effects were observed through UHR (β = 0.018, *P* < .001), FIB-4 (β = 0.004, *P* < .001), and RAR (β = 0.001, *P* = .040). TyG-BMI retained significant direct effects in models adjusting for UHR (β = 0.010, *P* < .001) and FIB-4 (β = 0.011, *P* < .001), but this effect was attenuated when adjusting for RAR (β = 0.006, *P* = .120). These results confirm that TyG-BMI promotes CKD risk via uric acid-lipid imbalance (UHR), hepatic fibrogenesis (FIB-4), and systemic inflammation (RAR), highlighting the multifactorial pathways relating metabolic dysfunction to renal deterioration in the CKM spectrum (Fig. 3).

## 4. Discussion

The CKM syndrome represents a pathophysiological continuum wherein multisystem interactions may critically influence chronic kidney disease (CKD) progression. In this context, our findings suggest that TyG-BMI, a composite marker of insulin resistance, appears to be associated with CKD progression across CKM stages. Our analysis further explores potential underlying mechanisms by examining the mediating roles of UHR, FIB-4, and RAR, revealing dose-response relationships between these metabolic indices and renal outcomes. Notably, UHR and FIB-4 showed progressively stronger associations with renal deterioration at advanced CKM stages (*P*-interaction < .005), which might support their potential utility as biomarkers for stage-specific risk stratification in high-risk CKM populations.

Mediation analysis revealed 3 potential pathways through which TyG-BMI influences CKD progression: uric acid dysregulation (UHR; accounting for 38.9% of the total effect), hepatic fibrosis (FIB-4; 22.2%), and systemic inflammation (RAR; 14.3%). Although TyG-BMI remained directly associated with CKD (*P* < .001), the majority of its impact was mediated indirectly via these pathways, with UHR identified as the most significant mediator. This finding underscores the potential of targeting uric acid metabolism as a therapeutic strategy. These results are consistent with previous research on the role of uric acid in kidney disease. Copur et al^[10]^ reported a strong association between elevated serum uric acid and accelerated renal function decline in patients with metabolic syndrome. Similarly, Sturm et al^[12]^ observed an independent relationship between uric acid levels and CKD progression in nondiabetic populations, though this association weakened after adjustment for renal function. Additionally, Kanbay et al^[15]^ identified hyperuricemia as an independent predictor of cardiovascular events in CKD patients. Our study supports these earlier findings while offering further mechanistic insight. The elevated UHR observed in patients with CKM syndrome appears to reflect a distinct “uric acid–metabolic imbalance” pathway, wherein uric acid interacts with other metabolic abnormalities – particularly high-density lipoprotein dysfunction – potentially exacerbating renal injury through synergistic mechanisms.

This study further elucidates the role of hepatic fibrosis in the TyG-BMI–renal dysfunction relationship within CKM syndrome. Our findings demonstrate a stage-dependent association between TyG-BMI and FIB-4 (β = 0.32, *P* < .001), with particular significance in advanced CKM stages. The mediation analysis indicated that 22.2% of TyG-BMI’s effect on CKD progression was mediated through FIB-4. These results extend previous observations by Xu et al^[11]^ in NAFLD patients and Seko et al^[13]^ in general populations, suggesting that TyG-BMI may promote renal impairment partly through hepatic fibrogenesis, potentially via profibrotic mediator release that exacerbates renal damage. The red cell distribution width-to-albumin ratio (RAR) has recently gained recognition as a prognostic biomarker in various disease states. Previous studies have established its clinical utility: Kimura et al^[14]^ demonstrated that elevated RAR levels were significantly associated with end-stage renal disease risk in CKD patients, while Liu et al^[16]^ reported its independent predictive value for cardiovascular mortality in acute myocardial infarction patients. Building upon this evidence, our study extends the understanding of RAR to the CKM syndrome context, revealing that it mediated 14.3% of the association between TyG-BMI and CKD risk (*P* = .006). This observation suggests that RAR might contribute to renal dysfunction through potential mechanisms involving both microvascular impairment and chronic systemic inflammation pathways.

This study represents a preliminary exploration of the potential mechanisms linking TyG-BMI to CKD risk within the theoretical framework of CKM syndrome. Cross-sectional analyses suggest possible multi-pathway associations between TyG-BMI and CKD risk, with UHR (uric acid metabolism), FIB-4 (hepatic fibrosis), and RAR (systemic inflammation) potentially contributing to this relationship. While TyG-BMI quartiles showed modest effect sizes (e.g., Q2 OR = 1.16, Q4 OR = 1.17 in fully adjusted model), the stronger and more consistent associations observed for UHR (Q4 OR = 2.06) and FIB-4 (Q4 OR = 5.47) suggest these mediators may represent more substantial contributors to CKD risk within the CKM context. These initial findings may offer some clues for understanding multi-organ interactions in CKM syndrome: uric acid metabolism abnormalities might involve crystal deposition and endothelial dysfunction, hepatic fibrosis could be related to profibrotic factor release, and systemic inflammation might affect renal function through microvascular injury pathways. Furthermore, these associations appear to exhibit certain bidirectional characteristics-TyG-BMI might influence renal function through these mediators, while renal impairment could potentially exacerbate metabolic dysregulation in return. These observations may provide preliminary insights for conceptualizing CKM syndrome as a dynamic pathophysiological network involving multiple systems and pathways.

This study has several limitations that warrant consideration. First, despite adjustment for multiple confounders, residual confounding may persist due to unmeasured variables such as dietary patterns, genetic predisposition, and environmental factors. Second, the generalizability of our findings may be limited as the NHANES cohort represents the US population, and external validation in other ethnic and geographic populations is needed. Third, the single-timepoint measurement of biomarkers prevents assessment of their dynamic changes over time and their longitudinal impact on disease progression. Fourth, while statistical models supported a linear association between TyG-BMI and CKD, more complex biological relationships might exist. Finally, the observational design precludes causal inference, and the observed associations require validation through intervention studies and stratification by key demographic factors.

## 5. Conclusions

Based on the findings from this cross-sectional investigation, TyG-BMI demonstrates associations with CKD risk through several potential pathways, including uric acid metabolism, hepatic fibrosis, and systemic inflammation. These observations lend support to the concept of multi-organ interactions within the CKM syndrome framework. Future research should focus on validating these associations in prospective cohorts, particularly in diverse populations. Additionally, studies exploring novel uric acid-related biomarkers and evaluating the efficacy of urate-lowering interventions in modifying CKD progression among CKM patients are warranted.

## Author contributions

**Conceptualization:** FuChun Zhang.

**Data curation:** Ping Han, Gang Fu, Shanshan Guo.

**Formal analysis:** Bin Zhao, Yuyang Liu, Xue Zhou.

**Methodology:** Bin Zhao, YunFei Guo.

**Writing – original draft:** Bin Zhao, YunFei Guo.

**Writing – review & editing:** Yuzhu Wang.

## Supplementary Material


