# Supplementary material for: TyG-BMI and risk of CKD progression: Mediation by oxidative stress, fibrosis, and metabolic dysfunction
Source: Medicine (Baltimore). 2026 Feb 28;105(9):e47465. doi: 10.1097/MD.0000000000047465 (PMC12956218; doi:10.1097/MD.0000000000047465)
Supplement: Supplementary file 1 [file medi-105-e47465-s001.doc]

Supplementary Material

Table S1 Covariates from NHANES 2007-2018.

| Variable Name | Variable ID | Years |
| --- | --- | --- |
| Neutrophil Count | LBDNENO | 2007-2018 |
| Lymphocyte Count | LBDLYMNO | 2007-2018 |
| Platelet Count (PLT) | LBXPLTSI | 2007-2018 |
| BMI | BMXBMI | 2007-2018 |
| Triglycerides (TG) | LBDSTRSI | 2007-2018 |
| Fasting Plasma Glucose (FPG) | RIDAGEYR | 2007-2018 |
| Age | RIDAGEYR | 2007-2018 |
| Aspartate Aminotransferase (AST) | LBXSASSI | 2007-2018 |
| Alanine Aminotransferase (ALT) | LBXSATSI | 2007-2018 |
| Uric Acid (UA) | LBXSUA | 2007-2018 |
| High-Density Lipoprotein (HDL) | LBDHDD | 2007-2018 |
| Urine Albumin | URXUMS | 2007-2018 |

Table S2 Staging of CKM syndrome.

| Stage | Definition | Criteria |
| --- | --- | --- |
| 0 | No CKM Risk Factors | All of the following: (1) Not overweight/obese (BMI < 25 kg/m² or < 23 kg/m² for Asians; waist circumference ≤88 cm/≤102 cm for women/men or ≤80 cm/≤90 cm for Asian women/men). (2) No metabolic risk factors (hypertension, hypertriglyceridemia [<135 mg/dL], metabolic syndrome, prediabetes, diabetes) or chronic kidney disease (CKD). |
| 1 | Excess or Dysfunctional Adipose Tissue | 1. BMI ≥25 kg/m² (or ≥23 kg/m² for Asians). 2. Waist circumference ≥88 cm/≥102 cm (women/men, or ≥80 cm/≥90 cm for Asians). 3. Presence of prediabetes (fasting glucose 100-124 mg/dL, HbA1c 5.7%-6.4%, or taking diabetes medications [to convert glucose to mmol/L, multiply by 0.0555; to convert HbA1c to the proportion of total hemoglobin, multiply by 0.01]). 4. No other metabolic risk factors or CKM |
| 2 | Metabolic Risk Factors and CKD | Presence of metabolic risk factors (triglycerides ≥135 mg/dL, hypertension, metabolic syndrome, diabetes) or moderate- to high-risk CKD. |
| 3 | Subclinical Cardiovascular Disease in CKM | Very high-risk CKD or 10-year cardiovascular disease risk ≥20% as predicted by the PREVENT risk model. |
| 4 | Clinical Cardiovascular Disease in CKM | Presence of clinical cardiovascular disease (coronary heart disease, angina, stroke, heart failure, myocardial infarction, or peripheral artery disease) |

Note: BMI = body mass index; CKD = chronic kidney disease; PREVENT = prediction of cardiovascular disease events.

Table S3 Variable definitions from NHANES 2007-2018.

| Variable Name | Variable ID | Definition | Years |
| --- | --- | --- | --- |
| Age | RIDAGEYR | Age in years at the time of household screening; individuals aged 85 or older are coded as 85. | 2007-2018 |
| Sex | RIAGENDR | Male or female. | 2007-2018 |
| Poverty-Income Ratio (PIR) | INDFMPIR | Ratio of family income to the poverty threshold. | 2007-2018 |
| Education Level | DMDHREDU | Highest grade or level of school completed. | 2007-2018 |
| Smoking Status | SMQ020 | Lifetime history of smoking at least 100 cigarettes. | 2007-2018 |
| Alcohol Consumption | ALQ101 | History of consuming at least 12 alcoholic drinks in a year. | 2007-2018 |
| Triglycerides (TG) | LBDSTRSI | Triglyceride levels (mmol/L). | 2007-2018 |
| Low-Density Lipoprotein (LDL) | LBDLDL | LDL-cholesterol levels (mg/dL). | 2007-2018 |
| High-Density Lipoprotein (HDL) | LBDHDD | Direct HDL-cholesterol levels (mg/dL). | 2007-2018 |
| Diabetes | DIQ010 | Physician-diagnosed diabetes. | 2007-2018 |

TableS4 Baseline characteristics of all participants.

|  |  | | level | 0 | 1 | 2 | 3 | 4 | P |
| --- | --- | --- | --- | --- | --- | --- | --- | --- | --- |
| n |  | |  | 1106 | 1333 | 3599 | 756 | 804 |  |
| gender (%) |  | | Male | 475 (42.9) | 560 (42.0) | 1778 (49.4) | 492 (65.1) | 468 (58.2) | <0.001 |
|  |  | | Female | 631 (57.1) | 773 (58.0) | 1821 (50.6) | 264 (34.9) | 336 (41.8) |  |
|  | | age (mean (SD)) | | 35.54 (13.68) | 39.02 (13.36) | 48.67 (14.93) | 70.64 (11.36) | 65.66 (12.88) | <0.001 |
| education (%) |  | | Less than 9th grade | 47 (4.2) | 95 (7.1) | 333 (9.3) | 134 (17.7) | 112 (13.9) | <0.001 |
|  |  | | 9-11th grade (Includes 12th grade with no diploma) | 122 (11.0) | 154 (11.6) | 559 (15.5) | 107 (14.2) | 143 (17.8) | |
|  |  | | High school graduate/GED or equivalent | 215 (19.4) | 290 (21.8) | 815 (22.6) | 214 (28.3) | 208 (25.9) | |
|  |  | | Some college or AA degree | 337 (30.5) | 418 (31.4) | 1081 (30.0) | 171 (22.6) | 202 (25.1) | |
|  |  | | College graduate or above | 385 (34.8) | 376 (28.2) | 811 (22.5) | 130 (17.2) | 139 (17.3) | |
|  | | PIR (mean (SD)) | | 2.70 (1.67) | 2.66 (1.65) | 2.55 (1.64) | 2.45 (1.48) | 2.25 (1.49) | <0.001 |
| alcohol (%) |  | | Yes | 836 (75.6) | 1007 (75.5) | 2596 (72.1) | 528 (69.8) | 573 (71.3) | 0.006 |
|  |  | | No | 270 (24.4) | 26 (24.5) | 1003 (27.9) | 228 (30.2) | 231 (28.7) |  |
| smoking (%) |  | | Yes | 396 (35.8) | 495 (37.1) | 1627 (45.2) | 481 (63.6) | 493 (61.3) | <0.001 |
|  |  | | No | 710 (64.2) | 838 (62.9) | 1972 (54.8) | 275 (36.4) | 311 (38.7) |  |
| diabetes (%) |  | | Yes | 0 (0.0) | 0 (0.0) | 412 (11.4) | 219 (29.0) | 238 (29.6) | <0.001 |
|  |  | | No | 1106 (100.0) | 1332 (99.9) | 3186 (88.5) | 404 (53.4) | 535 (66.5) |  |
|  |  | | Borderline | 0 (0.0) | 1 (0.1) | 1 (0.0) | 133 (17.6) | 31 (3.9) |  |
|  | | TG (mean (SD)) | | 0.82 (0.30) | 0.93 (0.30) | 1.63 (0.83) | 1.47 (0.77) | 1.53 (0.84) | <0.001 |
|  | | HDL (mean (SD)) | | 62.06 (15.57) | 56.04 (13.49) | 52.81 (16.75) | 51.85 (14.21) | 51.06 (15.70) | <0.001 |
|  | | LDL (mean (SD)) | | 103.13 (29.74) | 116.45 (32.45) | 120.81 (35.89) | 108.62 (35.02) | 100.71 (37.54) | <0.001 |
|  | | SII (mean (SD)) | | 475.34 (276.10) | 505.60 (289.32) | 539.74 (558.04) | 588.12 (430.55) | 584.87 (415.08) | <0.001 |
|  | | TyG-BMI (mean (SD)) | | 77.97 (12.08) | 111.75 (22.62) | 131.77 (36.20) | 128.74 (37.06) | 132.92 (40.31) | <0.001 |
|  | | FIB_4 (mean (SD)) | | 0.85 (0.69) | 0.89 (0.96) | 1.10 (0.81) | 1.87 (1.03) | 1.77 (1.05) | <0.001 |
|  | | UHR (mean (SD)) | | 8.19 (3.31) | 9.86 (3.69) | 11.80 (5.05) | 12.75 (5.33) | 13.12 (5.86) | <0.001 |
|  | | UACR (mean (SD)) | | 0.10 (0.16) | 0.09 (0.14) | 0.24 (1.08) | 1.96 (8.96) | 1.22 (5.32) | <0.001 |
|  | | eGFR (mean (SD)) | | 105.28 (16.98) | 101.86 (17.44) | 94.21 (18.99) | 66.96 (22.19) | 71.87 (22.67) | <0.001 |
| Depression (%) |  | | No | 1039 (93.9) | 1261 (94.6) | 3289 (91.4) | 699 (92.5) | 666 (82.8) | <0.001 |
|  |  | | Yes | 67 (6.1) | 72 (5.4) | 310 (8.6) | 57 (7.5) | 138 (17.2) |  |
|  | | RAR (mean (SD)) | | 2.95 (0.37) | 3.14 (0.48) | 3.15 (0.46) | 3.28 (0.49) | 3.32 (0.50) | <0.001 |
|  | | HGI (mean (SD)) | | -0.11 (0.35) | -0.08 (0.37) | 0.01 (0.66) | 0.13 (0.74) | 0.10 (0.80) | <0.001 |

|  | level | 1 | 2 | 3 | 4 | P |
| --- | --- | --- | --- | --- | --- | --- |
| n |  | 1901 | 5162 | 1074 | 1128 |  |
| gender (%) | Male | 789 (41.5) | 2555 (49.5) | 697 (64.9) | 652 (57.8) | <0.001 |
|  | Female | 1112 (58.5) | 2607 (50.5) | 377 (35.1) | 476 (42.2) |  |
| age (mean (SD)) |  | 38.99 (13.46) | 48.80 (15.00) | 70.55 (11.54) | 65.70 (12.91) | <0.001 |
| education (%) | Less than 9th grade | 132 (6.9) | 500 (9.7) | 177 (16.5) | 155 (13.7) | <0.001 |
|  | 9-11th grade (Includes 12th grade with no diploma) | 235 (12.4) | 792 (15.3) | 156 (14.5) | 200 (17.7) |  |
|  | High school graduate/GED or equivalent | 392 (20.6) | 1197 (23.2) | 284 (26.4) | 302 (26.8) |  |
|  | Some college or AA degree | 602 (31.7) | 1532 (29.7) | 258 (24.0) | 281 (24.9) |  |
|  | College graduate or above | 540 (28.4) | 1141 (22.1) | 199 (18.5) | 190 (16.8) |  |
| PIR (mean (SD)) |  | 2.66 (1.66) | 2.56 (1.64) | 2.48 (1.50) | 2.23 (1.49) | <0.001 |
| alcohol (%) | Yes | 1436 (75.5) | 3717 (72.0) | 739 (68.8) | 794 (70.4) | <0.001 |
|  | No | 465 (24.5) | 1445 (28.0) | 335 (31.2) | 334 (29.6) |  |
| smoking (%) | Yes | 705 (37.1) | 2321 (45.0) | 678 (63.1) | 690 (61.2) | <0.001 |
|  | No | 1196 (62.9) | 2841 (55.0) | 396 (36.9) | 438 (38.8) |  |
| diabetes (%) | Yes | 0 (0.0) | 603 (11.7) | 312 (29.1) | 351 (31.1) | <0.001 |
|  | No | 1899 (99.9) | 4558 (88.3) | 572 (53.3) | 732 (64.9) |  |
|  | Borderline | 2 (0.1) | 1 (0.0) | 190 (17.7) | 45 (4.0) |  |
| TG (mean (SD)) |  | 0.92 (0.30) | 1.63 (0.84) | 1.48 (0.78) | 1.51 (0.81) | <0.001 |
| HDL (mean (SD)) |  | 56.38 (13.85) | 52.71 (16.46) | 51.87 (14.24) | 51.26 (15.92) | <0.001 |
| LDL (mean (SD)) |  | 115.19 (31.35) | 120.69 (35.92) | 108.68 (35.10) | 101.21 (37.56) | <0.001 |
| SII (mean (SD)) |  | 507.43 (285.71) | 535.24 (494.75) | 574.75 (404.89) | 591.08 (420.15) | <0.001 |
| TyG_BMI (mean (SD)) |  | 111.24 (22.66) | 131.94 (36.34) | 128.54 (35.98) | 132.64 (39.28) | <0.001 |
| FIB_4 (mean (SD)) |  | 0.87 (0.84) | 1.10 (0.78) | 1.91 (1.14) | 1.78 (1.08) | <0.001 |
| UHR (mean (SD)) |  | 9.77 (3.75) | 11.79 (5.01) | 12.67 (5.27) | 13.11 (5.77) | <0.001 |
| UACR (mean (SD)) |  | 0.09 (0.14) | 0.24 (1.13) | 1.70 (8.09) | 1.03 (4.62) | <0.001 |
| eGFR (mean (SD)) |  | 101.96 (17.42) | 94.21 (19.08) | 67.59 (22.15) | 71.84 (22.97) | <0.001 |
| Depression (%) | No | 1800 (94.7) | 4725 (91.5) | 995 (92.6) | 951 (84.3) | <0.001 |
|  | Yes | 101 (5.3) | 437 (8.5) | 79 (7.4) | 177 (15.7) |  |
| RAR (mean (SD)) |  | 3.13 (0.47) | 3.15 (0.46) | 3.28 (0.48) | 3.33 (0.53) | <0.001 |
| HGI (mean (SD)) |  | -0.09 (0.37) | 0.01 (0.64) | 0.11 (0.71) | 0.13 (0.83) | <0.001 |

**Table S5** Association of metabolic indices with CKD (excluding participants with advanced CKD, n= 9172)

| Indices | Model 1 | | Model 2 | | Model 3 | |
| --- | --- | --- | --- | --- | --- | --- |
|  | OR (95%CI) | *P*-Value | OR (95%CI) | *P*-Value | OR (95%CI) | *P*-Value |
| TyG-BMI |  |  |  |  |  |  |
| Per unit | 1.00 (0.99, 1.00) | 0.328 | 1.00 (0.99, 1.00) | 0.237 | 1.00 (0.99, 1.01) | 0.984 |
| Q1 | Ref |  | Ref |  | Ref |  |
| Q2 | 1.15 (1.01, 1.32) | 0.039 | 1.13 (0.99, 1.30) | 0.077 | 1.16 (1.01, 1.34) | 0.034 |
| Q3 | 1.02 (0.89, 1.17) | 0.789 | 1.01 (0.88, 1.16) | 0.910 | 1.00 (0.87, 1.15) | 0.973 |
| Q4 | 1.17 (1.01, 1.34) | 0.030 | 1.17 (1.02, 1.35) | 0.025 | 1.10 (0.95, 1.26) | 0.207 |
| P trend | 1.00 (0.99, 1.00) | 0.110 | 1.00 (0.99, 1.00) | 0.079 | 1.01 (0.99, 1.01) | 0.546 |
| UHR |  |  |  |  |  |  |
| Per unit | 1.05 (1.04. 1.06) | < 0.001 | 1.05 (1.04, 1.06) | < 0.001 | 1.05 (1.03, 1.06) | < 0.001 |
| Q1 | Ref |  | Ref |  | Ref |  |
| Q2 | 1.37 (1.18, 1.58) | < 0.001 | 1.41 (1.22, 1.63) | < 0.001 | 1.41 (1.22, 1.64) | < 0.001 |
| Q3 | 1.50 (1.29, 1.74) | < 0.001 | 1.54 (1.32, 1.79) | < 0.001 | 1.50 (1.29, 1.75) | < 0.001 |
| Q4 | 2.00 (1.71, 2.34) | < 0.001 | 2.08 (1.77, 2.44) | < 0.001 | 2.03 (1.73, 2.38) | < 0.001 |
| P trend | 1.06 (1.05, 1.08) | < 0.001 | 1.07 (1.05, 1.08) | < 0.001 | 1.06 (1.05, 1.08) | < 0.001 |
| FIB-4 |  |  |  |  |  |  |
| Per unit | 1.64 (1.50, 1.79) | < 0.001 | 1.58 (1.45, 1.73) | < 0.001 | 1.51 (1.39, 1.65) | < 0.001 |
| Q1 | Ref |  | Ref |  | Ref |  |
| Q2 | 2.34 (1.91, 2.89) | < 0.001 | 2.20 (1.79, 2.72) | < 0.001 | 2.13 (1.73, 2.63) | < 0.001 |
| Q3 | 3.49 (2.87, 4.26) | < 0.001 | 3.18 (2.61, 3.90) | < 0.001 | 3.00 (2.46, 3.69) | < 0.001 |
| Q4 | 6.63 (5.44, 8.12) | < 0.001 | 6.01 (4.92, 7.38) | < 0.001 | 5.53 (4.52, 6.81) | < 0.001 |
| P trend | 3.73 (3.28, 4.25) | < 0.001 | 3.51 (3.08, 4.00) | < 0.001 | 3.30 (2.89, 3.77) | < 0.001 |
| RAR |  |  |  |  |  |  |
| Per unit | 1.07 (0.96, 1.18) | 0.231 | 1.12 (1.00, 1.24) | 0.041 | 1.10 (0.99, 1.23) | 0.073 |
| Q1 | Ref |  | Ref |  | Ref |  |
| Q2 | 1.07 (0.92, 1.23) | 0.376 | 1.07 (0.93, 1.24) | 0.365 | 1.08 (0.93, 1.25) | 0.304 |
| Q3 | 1.34 (1.16, 1.55) | < 0.001 | 1.38 (1.19, 1.59) | < 0.001 | 1.38 (1.20, 1.60) | < 0.001 |
| Q4 | 1.25 (1.08, 1.45) | 0.003 | 1.34 (1.16, 1.56) | < 0.001 | 1.32 (1.14, 1.54) | < 0.001 |
| P trend | 1.31 (1.12, 1.53) | < 0.001 | 1.43 (1.22, 1.67) | < 0.001 | 1.39 (1.19, 1.64) | < 0.001 |

**T****able S6 .** Association of TyG-BMI with UHR, FIB-4, and RAR indices

| Indices | Model 1 | | Model 2 | | Model 3 | |
| --- | --- | --- | --- | --- | --- | --- |
|  | β (95%CI) | *P*-Value | β (95%CI) | *P*-Value | β (95%CI) | *P*-Value |
| UHR | 3.58 (3.44, 3.72) | < 0.001 | 3.56 (3.42, 3.69) | < 0.001 | 3.41 (3.27, 3.55) | < 0.001 |
| FIB-4 | -2.95 (-3.81, -2.10) | < 0.001 | -3.05 (-3.91, -2.21) | < 0.001 | -3.75 (-4.58, -2.92) | < 0.001 |
| RAR | 14.4 (12.8, 15.9) | < 0.001 | 13.9 (12.3, 15.4) | < 0.001 | 12.2 (10.7, 13.7) | < 0.001 |

Model 1: adjusted for age, sex, and race

Model 2: further adjusted for marital status, education level, family poverty-income ratio (PIR), smoking status, and alcohol use

Model 3: further adjusted drugs for hypertension, diabetes, and depression status
